# Supplementary material for: No Additional Benefit of 185 Hz Versus 130 Hz at Equivalent Energy in Deep Brain Stimulation for Tremor: A Randomized Prospective Clinical Trial
Source: Mov Disord Clin Pract. 2026 Nov 2:10.1002/mdc3.70886. Online ahead of print. doi: 10.1002/mdc3.70886 (PMC13728422; doi:10.1002/mdc3.70886)
Supplement: Supplementary file 2 [file MDC3-9999-0-s002.docx]

## Supplementary Table S1: Amplitudes of TEED-equivalent Steps

|  | **Amplitudes 130 Hz [mA]** | **Amplitudes 185 Hz [mA]** | **TEED [µJ/s] (1000 Ohm, 60 µs, 130 Hz)** | **TEED [µJ/s] (1000 Ohm, 60 µs, 185Hz )** |
| --- | --- | --- | --- | --- |
| **1** | 0.5 | 0.4 | 2 | 1.8 |
| **2** | 1 | 0.8 | 7.8 | 7.1 |
| **3** | 1.2 | 1 | 11.2 | 11.1 |
| **4** | 2 | 1.7 | 31.2 | 32.1 |
| **5** | 2.4 | 2 | 44.9 | 44.4 |
| **6** | 3 | 2.5 | 70.2 | 69.4 |
| **7** | 3.6 | 3 | 101.1 | 99.9 |

***Supplementary Table S1: Amplitudes of TEED-equivalent Steps.*** Amplitudes of the two measurement series for ET (A for 130 Hz, B for 185 Hz; differences in TEED values result from rounding).

## Supplementary Material

## Accelerometry Model Parameters for Intention/Kinetic Tremor

Accelerometric assessment of intention tremor mainly followed the previously published Method in van der Linden et al. 2023 and 2026.^1,2^ The previously published model resulted in a numerical good fit for Intention Tremor (Finger [Wrist]: weighted kappa = 0.5 [0.4], percentage concordance of 67.4% [63.1%]). However, it resulted in a nearly linear relationship between mean Acceleration and predicted tremor score due to the low number of observations with higher clinical ratings. Therefor the model was adapted by weighting each value of clinical rating (0,1,2,3,4) by the number of observations. Numerically the new model resulted in lower weighted kappa and percentage concordance at lower clinical ratings, but a higher concordance at higher clinical ratings.

***Supplementary Figure S1:*** Illustration of the fit of the non-weighted (blue) and the weighted model (red) for accelerometric intention tremor ratings by finger accelerometry.

| **Model** | **Location** | **TR = a * T^c^ + b** | | | **RMSE** | **Weighted Kappa** | **Concordance** |
| --- | --- | --- | --- | --- | --- | --- | --- |
|  |  | **a** | **b** | **c** |  |  |  |
| non-weighted | Finger | 4.47 | -0.39 | 0.94 | 0.628 | 0.50 | 0.674 |
|  | Wrist | 8.91 | -0.29 | 1.11 | 0.917 | 0.40 | 0.631 |
| weighted | Finger | 10.6 | -6.6 | 0.16 | 0.085 | 0.15 | 0.409 |
|  | Wrist | 209.17 | -201.76 | 0.05 | 0.111 | 0.16 | 0.249 |

***Supplementary Table S2****:* **Accelerometry Model Parameters for Intention/Kinetic Tremor.** Model parameters (coefficients a, b, and c) determined in the training dataset, weighted Kappa and Concordance between clinical ratings and the resulting accelerometric tremor score when applying the model parameters to the validation data set. Abbreviations: *a* = slope, *b* = intercept, *c* = exponent of a power relationship between *T* and *TR*, RMSE = root mean squared error, T = tremor severity assessed by Mean Acceleration, TR = tremor rating score

## Supplementary Table S3: Patient Characteristics

|  | **ET** | **PD** |
| --- | --- | --- |
| **Cohort Size** | | |
| Patients | 18 (7 female) | 25 (6 female) |
| Hemispheres | 29 | 30 |
| Bilaterally tested | 11 | 5 |
| **Patient Characteristics** |  | |
| Age [years] (SD) | 69.3 (±7.7) | 61.2 (±7.5) |
| Hoehn and Yahr Stage | n. a. | 2.15 (±0.6) |
| Disease duration [years] (SD) | 26.2 (±15.4) | 9.3 (±3.6) |
| Time since surgery [months] (SD) | 38.1 (±23.8) | 28.4 (±20.4) |
| **Symptom Severity (ET: TRS, PD: UPDRS)** | | |
| Hemiscore MedOFF/StimOFF (SD) | 21.4 (±6.6) | 19.4 (±6.3) |
| Hemiscore MedOFF/StimONª (SD) | 7.7 (±4.8) | 7.6 (±4.5) |
| **DBS Leads** |  |  |
| Cartesia Directional Boston Scientific  Cartesia X Boston Scientific ^b^ | 26  0 | 27  1 |
| Medtronic Sensight B3300542 | 3 | 2 |
| Medtronic Sensight B3301542 | 1 | 0 |

^a^ Chronic stimulation settings

***Supplementary Table S3: Patient Characteristics.*** Abbreviations: DBS = deep brain stimulation, ET = Essential Tremor, MedOFF = after 12 hours withdrawal of disease-specific medication, PD = Parkinson’s Disease, SD = standard deviation, StimOFF = Stimulation OFF, StimON = Stimulation ON, TRS = Fahn Tolosa Marin Tremor Rating Scale, UPDRS = Unified Parkinson’s Disease Rating Scale

## Supplementary Table S4A: Contact Settings of the Essential Tremor Cohort

| 1[%] | 2[%] | 3[%] | 4[%] | 5[%] | 6[%] | 7[%] | 8[%] | Hemisphere | Lead Type |
| --- | --- | --- | --- | --- | --- | --- | --- | --- | --- |
| 0 | -34 | -33 | -33 | 0 | 0 | 0 | 0 | right | Vercise Cartesia |
| 0 | -100 | 0 | 0 | 0 | 0 | 0 | 0 | right | Vercise Cartesia |
| -100 | 0 | 0 | 0 | 0 | 0 | 0 | 0 | left | Vercise Cartesia |
| 0 | 0 | 0 | 0 | 0 | -80 | -60 | 0 | right | Vercise Cartesia |
| 0 | -50 | -50 | 0 | 0 | 0 | 0 | 0 | left | Vercise Cartesia |
| 0 | -34 | -33 | -33 | 0 | 0 | 0 | 0 | right | Vercise Cartesia |
| 0 | -34 | -33 | -33 | 0 | 0 | 0 | 0 | left | Sensight B3300542 |
| -50 | 0 | 0 | -50 | 0 | 0 | 0 | 0 | right | Vercise Cartesia |
| 0 | 0 | 0 | -80 | 0 | 0 | -20 | 0 | left | Vercise Cartesia |
| 0 | 0 | 0 | 0 | -70 | 0 | -30 | 0 | right | Vercise Cartesia |
| 0 | 0 | -25 | -75 | 0 | 0 | 0 | 0 | left | Vercise Cartesia |
| 0 | 0 | -50 | -50 | 0 | 0 | 0 | 0 | right | Vercise Cartesia |
| -20 | -20 | 0 | -60 | 0 | 0 | 0 | 0 | left | Vercise Cartesia |
| -40 | 0 | 0 | -60 | 0 | 0 | 0 | 0 | right | Vercise Cartesia |
| -100 | 0 | 0 | 0 | 0 | 0 | 0 | 0 | left | Vercise Cartesia |
| -100 | 0 | 0 | 0 | 0 | 0 | 0 | 0 | right | Vercise Cartesia |
| 0 | 0 | -100 | 0 | 0 | 0 | 0 | 0 | right | Vercise Cartesia |
| 0 | 0 | -100 | 0 | 0 | 0 | 0 | 0 | left | Vercise Cartesia |
| -100 | 0 | 0 | 0 | 0 | 0 | 0 | 0 | left | Vercise Cartesia |
| -60 | -14 | -13 | -13 | 0 | 0 | 0 | 0 | right | Vercise Cartesia |
| 0 | -34 | -33 | -33 | 0 | 0 | 0 | 0 | left | Sensight B3301542 |
| 0 | 0 | 0 | 0 | 0 | -40 | -60 | 0 | left | Sensight B3300542 |
| 0 | -34 | -33 | -33 | 0 | 0 | 0 | 0 | right | Sensight B3300542 |
| -100 | 0 | 0 | 0 | 0 | 0 | 0 | 0 | left | Vercise Cartesia |
| -100 | 0 | 0 | 0 | 0 | 0 | 0 | 0 | right | Vercise Cartesia |
| 0 | -34 | -33 | -33 | 0 | 0 | 0 | 0 | right | Vercise Cartesia |
| 0 | -34 | -33 | -33 | 0 | 0 | 0 | 0 | left | Vercise Cartesia |
| 0 | 0 | 0 | 0 | -10 | -80 | -10 | 0 | right | Vercise Cartesia |
| 0 | -34 | -33 | -33 | 0 | 0 | 0 | 0 | left | Vercise Cartesia |
| 0 | -24 | -23 | -23 | -10 | -10 | -10 | 0 | right | Vercise Cartesia |

## Supplementary Table S4A: Contact Settings of the Essential Tremor Cohort. Contact labeling was simplified to enable comparability across different lead types with contact 1 representing the most ventral and 8 the most dorsal contact. 2/3/4 and 5/6/7 correspond to the directional segments A/B/C counter-clockwise.

## Supplementary Table S4B: Contact Settings of the Parkinson’s Disease Cohort

| 1[%] | 2[%] | 3[%] | 4[%] | 5[%] | 6[%] | 7[%] | 8[%] | Hemisphere | Lead Type |
| --- | --- | --- | --- | --- | --- | --- | --- | --- | --- |
| 0 | 0 | 0 | -50 | 0 | 0 | -50 | 0 | right | Vercise Cartesia |
| 0 | 0 | 0 | 0 | 0 | -50 | 0 | -50 | left | Vercise Cartesia |
| 0 | 0 | 0 | 0 | -34 | -33 | -33 | 0 | left | Vercise Cartesia |
| 0 | 0 | -50 | 0 | 0 | -50 | 0 | 0 | left | Vercise Cartesia |
| 0 | 0 | 0 | 0 | -34 | -33 | -33 | 0 | left | Vercise Cartesia |
| 0 | 0 | 0 | 0 | 0 | -35 | -35 | -30 | right | Vercise Cartesia |
| 0 | 0 | 0 | 0 | 0 | -75 | -25 | 0 | right | Vercise Cartesia X |
| 0 | -10 | -10 | -10 | -23 | -23 | -24 | 0 | left | Vercise Cartesia |
| 0 | 0 | -70 | 0 | 0 | -30 | 0 | 0 | right | Vercise Cartesia |
| 0 | 0 | 0 | 0 | -34 | -33 | -33 | 0 | left | Vercise Cartesia |
| 0 | -10 | -10 | -10 | -22 | -24 | -24 | 0 | right | Vercise Cartesia |
| 0 | 0 | 0 | 0 | 0 | 0 | -40 | -60 | left | Vercise Cartesia |
| 0 | 0 | -30 | -30 | 0 | -20 | -20 | 0 | right | Vercise Cartesia |
| 0 | 0 | 0 | 0 | -100 | 0 | 0 | 0 | left | Vercise Cartesia |
| 0 | 0 | 0 | 0 | -50 | -50 | 0 | 0 | right | Vercise Cartesia |
| -40 | 0 | -15 | -45 | 0 | 0 | 0 | 0 | left | Vercise Cartesia |
| 0 | 0 | 0 | 0 | -24 | -23 | -23 | -30 | left | Vercise Cartesia |
| 0 | 0 | 0 | 0 | -34 | -33 | -33 | 0 | right | Vercise Cartesia |
| 0 | 0 | 0 | 0 | -34 | -33 | -33 | 0 | left | Vercise Cartesia |
| 0 | 0 | 0 | 0 | -30 | 0 | -30 | -40 | right | Vercise Cartesia |
| 0 | 0 | 0 | 0 | -100 | 0 | 0 | 0 | left | Vercise Cartesia |
| 0 | 0 | 0 | 0 | 0 | -25 | -75 | 0 | left | Vercise Cartesia |
| 0 | 0 | 0 | 0 | 0 | 0 | -40 | -60 | left | Vercise Cartesia |
| 0 | 0 | 0 | 0 | 0 | -25 | -75 | 0 | left | Vercise Cartesia |
| 0 | 0 | 0 | 0 | 0 | -25 | -75 | 0 | right | Vercise Cartesia |
| 0 | -50 | 0 | 0 | -50 | 0 | 0 | 0 | right | Sensight B3300542 |
| 0 | 0 | -100 | 0 | 0 | 0 | 0 | 0 | right | Vercise Cartesia |
| 0 | 0 | 0 | 0 | -75 | 0 | -25 | 0 | left | Vercise Cartesia |
| 0 | 0 | 0 | 0 | -100 | 0 | 0 | 0 | right | Vercise Cartesia |
| 0 | -34 | -33 | -33 | 0 | 0 | 0 | 0 | left | Sensight B3300542 |

## Supplementary Table S4B: Contact Settings of the Parkinson’s Disease Cohort. Contact labeling was simplified to enable comparability across different lead types with contact 1 representing the most ventral and 8 the most dorsal contact. 2/3/4 and 5/6/7 correspond to the directional segments A/B/C counter-clockwise.

## Supplementary Table S5: Linear Mixed Model Results (Secondary Outcomes)

| **Outcome** | **Fixed Effect** | **Estimate** | **95% CI [lower; upper]** | **p-value**  **(uncorr.)** | **p-value (FDR)** | **N** |
| --- | --- | --- | --- | --- | --- | --- |
| **Essential Tremor** | | | | | | |
| Postural Tremor  [ACC] | **Intercept** | **0.66** | **0.581; 0.74** | **<0.001** | **<0.001** | **300** |
|  | Frequency | -0.06 | -0.126; 0.006 | 0.073 | 0.499 | 300 |
|  | **TEED** | **0.005** | **0.004; 0.007** | **<0.001** | **<0.001** | **300** |
|  | Frequency:TEED | 0.001 | -0.001; 0.004 | 0.388 | >1 | 300 |
| Intention Tremor [ACC] | **Intercept** | **0.592** | **0.505; 0.678** | **<0.001** | **<0.001** | **300** |
|  | Frequency | -0.028 | -0.089; 0.033 | 0.374 | >1 | 300 |
|  | **TEED** | **0.007** | **0.006; 0.009** | **<0.001** | **<0.001** | **300** |
|  | Frequency:TEED | 0 | -0.003; 0.002 | 0.841 | >1 | 300 |
| Postural Tremor  [Clinical] | **Intercept** | **0.67** | **0.605; 0.736** | **<0.001** | **<0.001** | **301** |
|  | Frequency | -0.053 | -0.114; 0.009 | 0.095 | 0.499 | 301 |
|  | **TEED** | **0.006** | **0.005; 0.008** | **<0.001** | **<0.001** | **301** |
|  | Frequency:TEED | <0.001 | -0.002; 0.003 | 0.752 | >1 | 301 |
| Intention Tremor  [Clinical] | **Intercept** | **0.658** | **0.591; 0.725** | **<0.001** | **<0.001** | **301** |
|  | Frequency | -0.04 | -0.093; 0.012 | 0.131 | 0.499 | 301 |
|  | **TEED** | **0.007** | **0.006; 0.008** | **<0.001** | **<0.001** | **301** |
|  | Frequency:TEED | <0.001 | -0.002; 0.002 | 0.857 | >1 | 301 |
| Spiral [Clinical] | **Intercept** | **0.576** | **0.511; 0.64** | **<0.001** | **<0.001** | **301** |
|  | Frequency | <0.001 | -0.047; 0.047 | 0.994 | >1 | 301 |
|  | **TEED** | **0.006** | **0.005; 0.008** | **<0.001** | **<0.001** | **301** |
|  | Frequency:TEED | <0.001 | -0.002; 0.002 | 0.927 | >1 | 301 |
| **Parkinson’s Disease** | | | | | | |
| Resting Tremor  [ACC] | **Intercept** | **0.588** | **0.483; 0.692** | **<0.001** | **<0.001** | **381** |
|  | Frequency | -0.008 | -0.073; 0.057 | 0.808 | >1 | 381 |
|  | **TEED** | **0.006** | **0.005; 0.008** | **<0.001** | **<0.001** | **381** |
|  | Frequency:TEED | 0 | -0.002; 0.001 | 0.762 | >1 | 381 |
| Resting Tremor Provocation [ACC] | **Intercept** | **0.528** | **0.425; 0.63** | **<0.001** | **<0.001** | **381** |
|  | Frequency | 0.001 | -0.06; 0.062 | 0.972 | >1 | 381 |
|  | **TEED** | **0.006** | **0.005; 0.007** | **<0.001** | **<0.001** | **381** |
|  | Frequency:TEED | <0.001 | -0.002; 0.001 | 0.752 | >1 | 381 |
| Action Tremor [ACC] | **Intercept** | **0.502** | **0.353; 0.651** | **<0.001** | **<0.001** | **278** |
|  | Frequency | -0.036 | -0.105; 0.033 | 0.305 | >1 | 278 |
|  | **TEED** | **0.005** | **0.004; 0.006** | **<0.001** | **<0.001** | **278** |
|  | Frequency:TEED | 0.001 | -0.001; 0.002 | 0.55 | >1 | 278 |
| Resting Tremor  [Clinical] | **Intercept** | **0.603** | **0.502; 0.704** | **<0.001** | **<0.001** | **382** |
|  | Frequency | -0.024 | -0.096; 0.048 | 0.518 | >1 | 382 |
|  | **TEED** | **0.006** | **0.005; 0.008** | **<0.001** | **<0.001** | **382** |
|  | Frequency:TEED | <0.001 | -0.002; 0.002 | 0.906 | >1 | 382 |

| Resting Tremor Provocation [Clinical] | **Intercept** | **0.546** | **0.454; 0.639** | **<0.001** | **<0.001** | **382** |
| --- | --- | --- | --- | --- | --- | --- |
|  | Frequency | 0.008 | -0.051; 0.067 | 0.786 | >1 | 382 |
|  | **TEED** | **0.006** | **0.005; 0.007** | **<0.001** | **<0.001** | **382** |
|  | Frequency:TEED | <0.001 | -0.002; 0.001 | 0.855 | >1 | 382 |
| Action Tremor [Clinical] | **Intercept** | **0.578** | **0.466; 0.691** | **<0.001** | **<0.001** | **278** |
|  | Frequency | -0.023 | -0.087; 0.041 | 0.476 | >1 | 278 |
|  | **TEED** | **0.005** | **0.004; 0.006** | **<0.001** | **<0.001** | **278** |
|  | Frequency:TEED | <0.001 | -0.001; 0.002 | 0.791 | >1 | 278 |
| Spiral [Clinical] | **Intercept** | **0.715** | **0.586; 0.844** | **<0.001** | **<0.001** | **243** |
|  | Frequency | -0.02 | -0.103; 0.063 | 0.632 | >1 | 243 |
|  | **TEED** | **0.003** | **0.001; 0.004** | **<0.001** | **<0.001** | **243** |
|  | Frequency:TEED | <0.001 | -0.002; 0.002 | 0.746 | >1 | 243 |
| Akinetic-rigid Symptoms | **Intercept** | **0.562** | **0.497; 0.627** | **<0.001** | **<0.001** | **382** |
|  | Frequency | 0.021 | -0.024; 0.067 | 0.359 | >1 | 382 |
|  | **TEED** | **0.006** | **0.005; 0.007** | **<0.001** | **<0.001** | **382** |
|  | Frequency:TEED | -0.001 | -0.002; 0.001 | 0.299 | >1 | 382 |

## Supplementary Table S5: Linear Mixed Model Results (Secondary Outcomes). Results of the linear mixed effect models for tremor subscores, rated clinically and evaluated by accelerometery. We report fixed-effect estimates in relation to 130 Hz (β), 95% CIs, two-sided p-values (alpha = 0.05) and the number of observations (N). Abbreviations: ACC = accelerometry.

## Supplementary Table S6A: Pairwise Comparisons – Total Tremor

|  | **Comparison** | **Median Improvement 130 Hz [IQR] in %** | **Median Improvement**  **185 Hz [IQR] in %** | **p-Value**  **(uncorr.)** | **p-Value**  **(FDR)** | **N** |
| --- | --- | --- | --- | --- | --- | --- |
|  | | | | | | |
| **ET** | **TEED-Steps** |  |  |  |  |  |
|  | 1 | 23.1 [13-36.8] | 26.2 [8.9-41.6] | 0.854 | >1 | 29 |
|  | 2 | 52.6 [38.4-71.1] | 46.3 [31.1-60.4] | 0.151 | >1 | 28 |
|  | 3 | 72.7 [50.6-83.4] | 60.2 [39.8-77.5] | 0.003 | 0.064 | 28 |
|  | 4 | 100 [85.1-100] | 96.1 [72.5-100] | 0.583 | >1 | 25 |
|  | 5 | 99.1 [71.5-100] | 90.3 [72.1-100] | 0.365 | >1 | 16 |
|  | 6 | 100 [70.8-100] | 100 [83.4-100] | 0.813 | >1 | 11 |
|  | 7 | 83.5 [67-100] | 94.5 [73.5-100] | 1 | >1 | 4 |
|  | **Amplitudes** | | | | | |
|  | 1 mA | 54.6 [36.8-71.5] | 60.1 [37.9-76] | 0.755 | >1 | 27 |
|  | 2 mA | 93.8 [71.6-100] | 91.7 [76.2-100] | 0.898 | >1 | 18 |
|  | 3 mA | 90.8 [62.6-100] | 100 [79.5-100] | 0.25 | >1 | 7 |
|  | | | | | | |
| **PD** | **TEED-Steps** |  |  |  |  |  |
|  | 1 | 10.1 [-1.5-22.2] | 6 [-8.6-24.4] | 0.055 | 0.594 | 28 |
|  | 2 | 31.6 [7.8-70.1] | 38.6 [4-61.3] | 0.909 | >1 | 30 |
|  | 3 | 40.5 [0.7-88.1] | 45.3 [8.8-93] | 0.925 | >1 | 30 |
|  | 4 | 70.8 [57.9-98.8] | 80.8 [37.5-100] | 0.831 | >1 | 28 |
|  | 5 | 77.4 [27.1-100] | 89.5 [66.9-100] | 0.911 | >1 | 26 |
|  | 6 | 100 [56.4-100] | 100 [61.2-100] | 0.455 | >1 | 25 |
|  | 7 | 100 [66.2-100] | 100 [47.1-100] | 0.195 | >1 | 19 |
|  | **Amplitudes** |  |  |  |  |  |
|  | 1 mA | 31.6 [7.8-70.1] | 45.3 [8.8-93] | 0.292 | 0.803 | 30 |
|  | 2 mA | 68.7 [57.8-98.4] | 89.5 [67.5-100] | 0.045 | 0.246 | 27 |
|  | 3 mA | 100 [69.6-100] | 100 [49.5-100] | 0.557 | >1 | 21 |

***Supplementary Table S6A: Pairwise Comparisons – Total Tremor.*** Abbreviations: ET = Essential Tremor, IQR = Interquartile Range,PD = Parkinson’s Disease.

## Supplementary Table S6B: Pairwise Comparisons – Total Tremor Clinical Rating

|  | **Comparison** | **Median Improvement 130 Hz [IQR] in %** | **Median Improvement**  **185 Hz [IQR] in %** | **p-Value**  **(uncorr.)** | **p-Value**  **(FDR)** | **N** |
| --- | --- | --- | --- | --- | --- | --- |
| **ET** | **TEED-Steps** | | | | | |
|  | 1 | 30 [14.3-39.6] | 26.7 [14.8-37] | 0.563 | >1 | 29 |
|  | 2 | 57.1 [41.7-68.8] | 55 [36.5-64.9] | 0.19 | >1 | 29 |
|  | 3 | 73.6 [62.7-80.6] | 64.6 [52.1-80] | 0.033 | 0.726 | 28 |
|  | 4 | 92.9 [71.9-97.3] | 93.3 [76-100] | 0.988 | >1 | 25 |
|  | 5 | 92.3 [73.9-93.8] | 87.5 [77.1-94.4] | 0.637 | >1 | 16 |
|  | 6 | 92.9 [82.5-95.8] | 95 [85.6-96.5] | 0.43 | >1 | 11 |
|  | 7 | 86.4 [71.8-96.1] | 84.2 [71-95.8] | 0.875 | >1 | 4 |
|  | **Amplitudes** | | | | | |
|  | 1 mA | **57.1 [41.7-66.7]** | **64.6 [52.1-80]** | **0.003** | **0.016** | **28** |
|  | 2 mA | 91.4 [62.5-95] | 88.8 [79.2-96.4] | 0.055 | 0.15 | 18 |
|  | 3 mA | 91.7 [71.9-95.6] | 91.7 [78.8-98.8] | 0.188 | 0.344 | 7 |
|  | | | | | | |
| **PD** | **TEED-Steps** |  |  |  |  |  |
|  | 1 | 11.1 [0-19.4] | 10.6 [0-33.3] | 0.289 | >1 | 28 |
|  | 2 | 34.6 [6.7-75] | 33.3 [14.3-66.7] | 0.695 | >1 | 30 |
|  | 3 | 56.9 [22.2-87.5] | 48.1 [23.1-91.7] | 0.538 | >1 | 30 |
|  | 4 | 81.7 [56.3-95.8] | 83.3 [48.3-100] | 0.794 | >1 | 28 |
|  | 5 | 80 [54.4-100] | 91.7 [64.4-100] | 0.777 | >1 | 27 |
|  | 6 | 100 [74.1-100] | 100 [71.3-100] | 0.445 | >1 | 25 |
|  | 7 | 100 [81.9-100] | 100 [63.8-100] | 0.266 | >1 | 19 |
|  | **Amplitudes** |  |  |  |  |  |
|  | 1 mA | 34.6 [6.7-75] | 48.1 [23.1-91.7] | 0.042 | 0.231 | 30 |
|  | 2 mA | 80 [56-97.9] | 91.7 [64.4-100] | 0.125 | 0.344 | 27 |
|  | 3 mA | 100 [74.1-100] | 100 [71.3-100] | 0.447 | 0.82 | 21 |

***Supplementary Table S6B: Pairwise Comparisons – Total Tremor Clinical Rating.*** Abbreviations: ET = Essential Tremor, IQR = Interquartile Range,PD = Parkinson’s Disease.

## Supplementary Table S7: Pairwise Comparisons – Subscores

| **Outcome** | **Condition** | **Median Improvement 130 Hz [IQR]** | **Median Improvement**  **185 Hz [IQR]** | **p-Value**  **(uncorr.)** | **p-Value**  **(FDR)** | **N** |
| --- | --- | --- | --- | --- | --- | --- |
| **ET** |  | | | | | |
| Postural Tremor [ACC] Improvement [%] | TEED Step 3 | 85.5 [50.5-100] | 62.5 [33.3-97.8] | 0.002 | 0.047 | 28 |
| **PD** |  | | | | | |
| Akinetic-rigid Symptoms  [Clinical] Improvement [%] | 1mA | 25 [14.3-50] | 50 [37.5-75] | <0.001 | <0.001 * | 30 |
| Akinetic-rigid Symptoms  [Clinical] Improvement [%] | 2mA | 66.7 [50-87.5] | 80 [71.4-100] | 0.002 | 0.006 * | 27 |

***Supplementary Table S7: Pairwise Comparisons –Subscores.*** Only significant differences are shown.

| **Metric** | **Fixed Effect** | **Estimate** | **95% CI [lower; upper]** | **p-value** | **N** |
| --- | --- | --- | --- | --- | --- |
| **Essential Tremor – Postural Tremor** | | | | | |
| Mean Acceleration | **Intercept** | **0.811** | **0.733; 0.888** | **<0.001** | **300** |
|  | Frequency | -0.05 | -0.114; 0.014 | 0.127 | 300 |
|  | **TEED** | **0.004** | **0.003; 0.006** | **<0.001** | **300** |
|  | Frequency:TEED | 0.001 | -0.002; 0.003 | 0.637 | 300 |
| Mean Envelope | **Intercept** | **0.811** | **0.734; 0.888** | **<0.001** | **300** |
|  | Frequency | -0.052 | -0.117; 0.012 | 0.109 | 300 |
|  | **TEED** | **0.004** | **0.003; 0.006** | **<0.001** | **300** |
|  | Frequency:TEED | 0.001 | -0.002; 0.003 | 0.600 | 300 |
| AUC Power | **Intercept** | **0.868** | **0.758; 0.978** | **<0.001** | **300** |
|  | Frequency | -0.081 | -0.185; 0.023 | 0.127 | 300 |
|  | **TEED** | **0.003** | **0.001; 0.006** | **0.012** | **300** |
|  | Frequency:TEED | 0.001 | -0.002; 0.005 | 0.518 | 300 |
| Peak Power | **Intercept** | **0.868** | **0.76; 0.976** | **<0.001** | **300** |
|  | Frequency | -0.071 | -0.168; 0.026 | 0.151 | 300 |
|  | **TEED** | **0.003** | **0.001; 0.006** | **0.010** | **300** |
|  | Frequency:TEED | 0.001 | -0.002; 0.004 | 0.549 | 300 |
| **Parkinson’s Disease – Rest Tremor** | | | | | |
| Mean Acceleration | **Intercept** | **0.601** | **0.485; 0.718** | **<0.001** | **381** |
|  | Frequency | -0.001 | -0.075; 0.074 | 0.988 | 381 |
|  | **TEED** | **0.006** | **0.005; 0.008** | **<0.001** | **381** |
|  | Frequency:TEED | 0 | -0.002; 0.002 | 0.678 | 381 |
| Mean Envelope | **Intercept** | **0.598** | **0.479; 0.717** | **<0.001** | **381** |
|  | Frequency | -0.002 | -0.077; 0.073 | 0.961 | 381 |
|  | **TEED** | **0.006** | **0.005; 0.008** | **<0.001** | **381** |
|  | Frequency:TEED | 0 | -0.002; 0.002 | 0.671 | 381 |
| AUC Power | **Intercept** | **0.55** | **0.334; 0.766** | **<0.001** | **381** |
|  | Frequency | -0.051 | -0.22; 0.119 | 0.558 | 381 |
|  | **TEED** | **0.009** | **0.006; 0.012** | **<0.001** | **381** |
|  | Frequency:TEED | 0.003 | -0.002; 0.007 | 0.267 | 381 |
| Peak Power | **Intercept** | **0.536** | **0.295; 0.777** | **<0.001** | **381** |
|  | Frequency | -0.068 | -0.261; 0.124 | 0.485 | 381 |
|  | **TEED** | **0.01** | **0.006; 0.013** | **<0.001** | **381** |
|  | Frequency:TEED | 0.003 | -0.002; 0.008 | 0.221 | 381 |

## Supplementary Table S8A: Linear mixed model for accelerometry metrics

***Supplementary Table S8A: Linear mixed model for accelerometry metrics.*** Results of the linear mixed effect models for accelerometry metrics. We report fixed-effect estimates in relation to 130 Hz (β), 95% CIs, two-sided p-values (alpha = 0.05) and the number of observations

## Supplementary Table 8B: Pairwise Comparisons – Accelerometry metrics for postural tremor in ET

| **TEED-Steps** | **Median Improvement 130 Hz [IQR]** | **Median Improvement**  **185 Hz [IQR]** | **p-Value**  **(uncorr.)** | **p-Value**  **(FDR)** |
| --- | --- | --- | --- | --- |
| **ET Postural Tremor – Mean Acceleration [g]** | | | | |
| 1 | 0.13 [0.05-0.33] | 0.16 [0.03-0.3] | 0.393 | >1 |
| 2 | 0.05 [0.02-0.14] | 0.04 [0.01-0.1] | 0.028 | 0.254 |
| 3 | 0.03 [0.01-0.1] | 0.02 [0-0.05] | 0.003 | 0.054 |
| 4 | 0 [0-0.01] | 0 [0-0.02] | 0.461 | >1 |
| 5 | 0 [0-0.02] | 0 [0-0.02] | 0.375 | >1 |
| 6 | 0 [0-0] | 0 [0-0.02] | 0.625 | >1 |
| 7 | 0 [0-0.03] | 0 [0-0.02] | 1 | >1 |
| **ET Postural Tremor – Mean Envelope [g]** | | | | |
| 1 | 0.29 [0.13-0.7] | 0.35 [0.06-0.68] | 0.43 | >1 |
| 2 | 0.12 [0.05-0.32] | 0.1 [0.02-0.21] | 0.032 | 0.290 |
| 3 | 0.06 [0.01-0.22] | 0.04 [0-0.1] | 0.003 | 0.054 |
| 4 | 0 [0-0.02] | 0 [0-0.04] | 0.461 | >1 |
| 5 | 0 [0-0.05] | 0 [0-0.04] | 0.469 | >1 |
| 6 | 0 [0-0] | 0 [0-0.05] | 0.625 | >1 |
| 7 | 0 [0-0.07] | 0 [0-0.05] | 1 | >1 |
| **ET Postural Tremor – AUC Power [mg^2^]** | | | | |
| 1 | 18.49 [3.56-98.68] | 26.55 [1.17-100.12] | 0.469 | >1 |
| 2 | 2.81 [0.48-27.42] | 1.63 [0.12-8.84] | 0.043 | 0.390 |
| 3 | 0.98 [0.05-11.07] | 0.37 [0-3.215] | 0.007 | 0.127 |
| 4 | 0 [0-0.11] | 0 [0-0.41] | 0.547 | >1 |
| 5 | 0 [0-0.52] | 0 [0-0.22] | 0.469 | >1 |
| 6 | 0 [0-0] | 0 [0-0.60] | 0.875 | >1 |
| 7 | 0 [0-1.77] | 0 [0-1.04] | 1 | >1 |
| **ET Postural Tremor – Peak Power [mg^2^/Hz]** | | | | |
| 1 | 6521.06 [570.39-30621.02] | 8025.91 [298.2-35990.24] | 0.482 | >1 |
| 2 | 608.03 [100.53-8163.05] | 520.14 [31.65-2580.32] | 0.049 | 0.445 |
| 3 | 225.26 [10.48-3381.91] | 90.83 [0-852.33] | 0.006 | 0.109 |
| 4 | 0 [0-23.69] | 0 [0-69.36] | 0.383 | >1 |
| 5 | 0 [0-104.15] | 0 [0-57.63] | 0.469 | >1 |
| 6 | 0 [0-0] | 0 [0-99.64] | 0.875 | >1 |
| 7 | 0 [0-498.4] | 0 [0-493.4] | 1 | >1 |

***Supplementary Table 8B: Pairwise Comparisons – Accelerometry metrics for postural tremor in ET.***

## Supplementary Table 8C: Pairwise Comparisons – Accelerometry metrics for resting tremor in PD

| **TEED-Steps** | **Median Improvement 130 Hz [IQR]** | **Median Improvement**  **185 Hz [IQR]** | **p-Value**  **(uncorr.)** | **p-Value**  **(FDR)** |
| --- | --- | --- | --- | --- |
| **PD Resting Tremor – Mean Acceleration [g]** | | | | |
| 1 | 0.51 [0.23-1.12] | 0.43 [0.12-1.17] | 0.885 | >1 |
| 2 | 0.13 [0.01-0.44] | 0.16 [0.01-0.77] | 0.292 | >1 |
| 3 | 0.07 [0-0.53] | 0.12 [0-0.58] | 0.263 | >1 |
| 4 | 0.03 [0-0.25] | 0.02 [0-0.1] | 0.958 | >1 |
| 5 | 0 [0-0.02] | 0.01 [0-0.13] | 0.124 | >1 |
| 6 | 0 [0-0.02] | 0 [0-0.02] | 0.193 | >1 |
| 7 | 0 [0-0.04] | 0 [0-0] | 0.297 | >1 |
| **PD Resting Tremor – Mean Envelope [g]** | | | | |
| 1 | 1.12 [0.51-2.41] | 0.95 [0.28-2.53] | 0.829 | >1 |
| 2 | 0.3 [0.02-0.94] | 0.36 [0.03-1.61] | 0.209 | >1 |
| 3 | 0.16 [0-1.18] | 0.27 [0-1.29] | 0.277 | >1 |
| 4 | 0.06 [0-0.56] | 0.04 [0-0.23] | 0.986 | >1 |
| 5 | 0.01 [0-0.04] | 0.01 [0-0.27] | 0.124 | >1 |
| 6 | 0 [0-0.05] | 0 [0-0.05] | 0.193 | >1 |
| 7 | 0 [0-0.11] | 0 [0-0] | 0.469 | >1 |
| **PD Resting Tremor – AUC Power [mg^2^]** | | | | |
| 1 | 197.59 [60.89-1153.84] | 154.30 [21.55-1049.60] | 0.4 | >1 |
| 2 | 20.41 [0.03-167.98] | 35.52 [0.12-479.70] | 0.166 | >1 |
| 3 | 7.02 [0-247.85] | 13.76 [0-331.26] | 0.485 | >1 |
| 4 | 0.58 [0-76.56] | 0.21 [0-14.51] | 0.931 | >1 |
| 5 | 0.02 [0-0.49] | 0.06 [0-14.24] | 0.093 | >1 |
| 6 | 0 [0-0.37] | 0 [0-0.52] | 0.375 | >1 |
| 7 | 0 [0-13.56] | 0 [0-0] | 0.297 | >1 |
| **PD Resting Tremor – Peak Power [mg^2^/Hz]** | | | | |
| 1 | 44426 [17214-393263] | 42670 [6346-398668] | 0.374 | >1 |
| 2 | 4751.83 [3.82-54536.86] | 6431.71 [28.6-167253.12] | 0.166 | >1 |
| 3 | 1858.64 [0-63059.97] | 3378.75 [0-100013.32] | 0.527 | >1 |
| 4 | 128.45 [0-24863.67] | 53.8 [0-4427.37] | 0.689 | >1 |
| 5 | 3.47 [0-98.31] | 7.73 [0-4729.01] | 0.076 | >1 |
| 6 | 0 [0-98.9] | 0 [0-147.18] | 0.16 | >1 |
| 7 | 0 [0-2864.76] | 0 [0-0] | 0.297 | >1 |

***Supplementary Table 8C: Pairwise Comparisons – Accelerometry metrics for postural tremor in PD.***

## Supplementary Table S9: Side Effect Comparisons

| **Data-set** | **Outcome** | **Median 130 Hz [IQR]** | **Median 185 Hz [IQR]** | **p-Value (uncorr.)** | **p-Value**  **(FDR)** | **N** |
| --- | --- | --- | --- | --- | --- | --- |
| **ET** | | | | | | |
|  | Max. Total Tremor Improvement [%] | 100 [100-100] | 100 [98-100] | 0.078 | 0.389 | 29 |
|  | **SE Amplitude [mA]** | **3 [2.5-3.5]** | **2.5 [2.2-3.1]** | **<0.001** | **0.001** | **29** |
|  | SE TEED [µJ/s] | 78 [39-116] | 67 [43-107] | 0.854 | >1 | 29 |
| **PD** | | | | | | |
|  | Max. Total Tremor Improvement [%] | 100 [96-100] | 100 [74-100] | 0.007 | 0.389 | 30 |
|  | **SE Amplitude [mA]** | **4.5 [3.5-5]** | **3.5 [3-4.5]** | **<0.001*** | **0.001** | **30** |
|  | SE TEED [µJ/s] | 134 [88-243] | 137 [78-274] | 0.049 | >1 | 30 |

***Supplementary Table S9: Side Effects.*** Maximal total Tremor Improvement 0.5 mA below the individual side-effect (SE) threshold. Abbreviations: SE = Side-effect, TEED = total electrical energy delivered.

## Supplementary Table S10: Side Effects

| **Side Effect** | **ET** | | **PD** | |
| --- | --- | --- | --- | --- |
|  | **130 Hz** | **185 Hz** | **130 Hz** | **185 Hz** |
| Dysarthria | 17 | 19 | 13 | 12 |
| Ataxia | 10 | 11 | - | - |
| Paresthesia | 5 | 3 | 5 | 10 |
| Dizziness | 3 | 2 | 2 | 2 |
| Troubled Vision | 3 | 2 | - | - |
| Capsule | 1 | 1 | 9 | 10 |
| Unspecific Discomfort | - | - | 2 | 2 |
| Dyskinesia | - | - | 1 | - |

***Supplementary Table S10: Observed Side Effects*** for ET and PD with both stimulation frequencies. The character of the side-effect caused by 130Hz and 185Hz respectively only differed in 5 of 59 hemispheres. In 8 of 59 hemispheres patients experienced the same side-effect but with a different additional side-effect, e.g. dysarthria with 130 Hz and 185 Hz but accompanied by paresthesia with 185 Hz.

Abbreviations: ET = Essential Tremor, PD = Parkinson’s Disease***.***

## Supplementary Figure S2: Lead Positions

***Supplementary Figure S2: Lead Positions in MNI ICBM 2009b space, illustrated with relevant anatomical structures.*** Postoperative CT images (IQon Spectral CT, iCT 256, Brilliance 256, Philips Healthcare, Best, The Netherlands) were linearly co-registered to preoperative magnetic resonance imaging (3-Tesla Philips Ingenia® Scanner, Philips, Amsterdam, The Netherlands) using advanced normalization tools (ANTs).^3^ A brain shift correction step was applied as implemented in Lead-DBS. Then, images were non-linearly normalized into standard space using ANTs and the “Effective: low variance, Default” setting. The results of each pre-processing step were visually inspected regarding accuracy and refined if necessary. DBS leads were automatically reconstructed with the PaCER algorithm,^4^ and manually refined. The orientation of the directional leads was determined using DiODE^5^ (A) Essential Tremor Cohort (B) Parkinson’s Disease Cohort Abbreviations: STN = Subthalamic Nucleus, VIM = ventral intermediate nucleus.

## Supplementary Figure S3: Individual Trajectories of Tremor Suppression

***Supplementary Figure S3: Individual Trajectories of Tremor Suppression.*** Illustration of individual trajectories of differences in tremor suppression between both frequency settings for the primary outcome across the testing. Each line represents an individual investigated hemisphere in (A) the Essential Tremor and (B) the Parkinson’s Disease cohort.

## References

1.	Linden C van der, Berger T, Dembek TA, et al. Tractography-guided versus clinical contact selection for deep brain stimulation in tremor - A prospective clinical trial [Internet]. Brain Stimul. Basic Transl. Clin. Res. Neuromodulation 2026;19(2)[cited 2026 Mar 6 ] Available from: https://www.brainstimjrnl.com/article/S1935-861X(26)00038-0/fulltext

2.	van der Linden C, Berger T, Brandt GA, et al. Accelerometric Classification of Resting and Postural Tremor Amplitude. Sensors 2023;23(20)

3.	Avants BB, Tustison NJ, Song G, et al. A reproducible evaluation of ANTs similarity metric performance in brain image registration. NeuroImage 2011;54(3):2033–2044.

4.	Husch A, V. Petersen M, Gemmar P, et al. PaCER - A fully automated method for electrode trajectory and contact reconstruction in deep brain stimulation. NeuroImage Clin. 2018;17:80–89.

5.	Dembek TA, Hoevels M, Hellerbach A, et al. Directional DBS leads show large deviations from their intended implantation orientation. Parkinsonism Relat. Disord. 2019;67:117–121.
